# Anatomical Relationship of Lateral Low-to-Low Nasal Osteotomy and Inferior Nasal Turbinate. Is Webster's Triangle Still Important?

**DOI:** 10.1055/a-2836-2147

**Published:** 2026-05-29

**Authors:** Atchara Setthajindalert, Padcha Tunlayadechanont, Thiti Tantitham

**Affiliations:** 1 Division of Plastic and Maxillofacial Surgery, Department of Surgery, Ramathibodi Hospital, Mahidol University, Bangkok, Thailand; 2 Division of Neuroimaging, Department of Diagnostic and Therapeutic Radiology, Ramathibodi Hospital, Mahidol University, Bangkok, Thailand

**Keywords:** rhinoplasty, nasal osteotomy, Webster's triangle, East Asian nasal anatomy, CT analysis, nasal turbinate, lateral osteotomy

## Abstract

**Background:**

Lateral low-to-low osteotomies in rhinoplasty raise safety concerns regarding Webster's triangle and airway patency. This study aimed to measure the distance between the osteotomy line and the anterior inferior nasal turbinate and evaluate its relationship with other nasal characteristics.

**Methods:**

We retrospectively analyzed CT scans from 81 East Asian patients (January–December 2020). This study used a virtual simulation to measure the distance between a projected lateral low-to-low osteotomy line and the anterior inferior nasal turbinate on CT scans, along with nasal bone length/thickness, pyriform aperture width, and rhinion–nasomaxillary suture distance. Linear regression and Spearman correlation assessed parameter associations.

**Results:**

The 81 patients (mean age 46.7 years) showed an average distance of 7.1 ± 3.2 mm (right) and 6.5 ± 3.0 mm (left) between the osteotomy line and the inferior nasal turbinate. A significant negative correlation ( *r* = −0.35, *p* < 0.001) was found between rhinion–nasomaxillary suture distance and this osteotomy–turbinate distance. No correlation was seen with nasal bone length or pyriform aperture width. Measurements demonstrated good intrarater reliability (intraclass correlation coefficient [ICC] = 0.98).

**Conclusions:**

The lateral low-to-low osteotomy line does not intersect the anterior nasal turbinate, indicating it can be safely performed without concern for Webster's triangle. However, caution is advised for patients with higher nasal dorsum projection (shorter rhinion–nasomaxillary suture distance). This study offers valuable insights into East Asian nasal characteristics for rhinoplasty.

## Introduction

Surgical anatomy is a fundamental aspect of all surgical procedures, and numerous studies have described nasal anatomy. However, the nasal characteristics of East Asians differ significantly from those of Caucasians, typically featuring shorter, flatter noses with broader nasal bones. 1 2 Rhinoplasty with nasal osteotomy is often necessary to correct these broader noses. 3 Lateral nasal osteotomy is a preferred procedure, typically performed in a high-to-low fashion to avoid injury to Webster's triangle.

Webster et al described Webster's triangle as a triangular piece of bone at the pyriform aperture, which should be preserved during low lateral osteotomy to maintain airway patency. They also recommended using curved or angulated lateral osteotomies. 4 In contrast, Gubisch argued that avoiding Webster's triangle may not be necessary, as medial displacement of the inferior turbinate head was not observed with percutaneous lateral low-to-low osteotomy. 5 Additionally, Guyuron suggested that patients with shorter nasal bones exhibit less airway reduction than those with longer nasal bones. 6

Nasal osteotomy is invasive, irreversible, and difficult to control. Procedures performed along straight lines are generally easier to control than those performed along curved or angulated lines. This study aimed to examine the distance between the lateral low-to-low osteotomy line and the anterior inferior nasal turbinate in East Asian adult rhinoplasty by quantitatively analyzing data from CT scans. Secondary outcomes included analyzing the relationship between this distance and other nasal characteristics, as well as identifying potential predictors for this distance based on other variables.

## Methods

### Materials

Between January and December 2020, CT scans of the sinus and paranasal sinuses from 81 Thai patients were analyzed using three-dimensional computed tomography. The study was conducted as a retrospective review of existing data and did not require informed consent. The hospital IRB approved the study (protocol number MURA2021/376). Informed consent for image use was obtained from the patients. Inclusion criteria were patients aged 18 years or older who had undergone sinus and paranasal sinus CT scans. Exclusion criteria included poor-quality CT scans, a history of nasal or nasal cavity surgery, nasal fractures, tumors, or infections that could distort the area of interest. Data collected included sex, age, race, the distance between the inferior nasal turbinate and the lateral low-to-low osteotomy path, lateral nasal bone thickness, nasal bone length, width of the pyriform aperture, and rhinion–nasomaxillary suture distance. To assess intrarater consistency, the same plastic surgeon repeated the measurements three times over 3 months.

### Computed Tomography Paranasal Sinus Protocol

The CT scans were performed with patients in the supine position, with the scout film perpendicular to the hard palate. The tube voltage and current were set at 125 kV and 80 to 160 mA, respectively. The scanning range extended from the hard palate to the top of the frontal sinuses. Scan parameters included a field of view of 140 to 160 mm and a slice thickness of 0.5 to 1.0 mm. Multiplanar reconstructions (axial, coronal, and sagittal) were used. 7 For this study, diagnostic measurements were done using an Acer B243PWL LED LCD monitor, compliant with the American Association of Physicists in Medicine (AAPM) and the Society for Imaging Informatics in Medicine (SIIM) standards.

### Virtual Simulation and Radiological Measurements: Distance between Inferior Nasal Turbinate and Lateral Low-to-Low Nasal Osteotomy Path

**Fig. 1:**
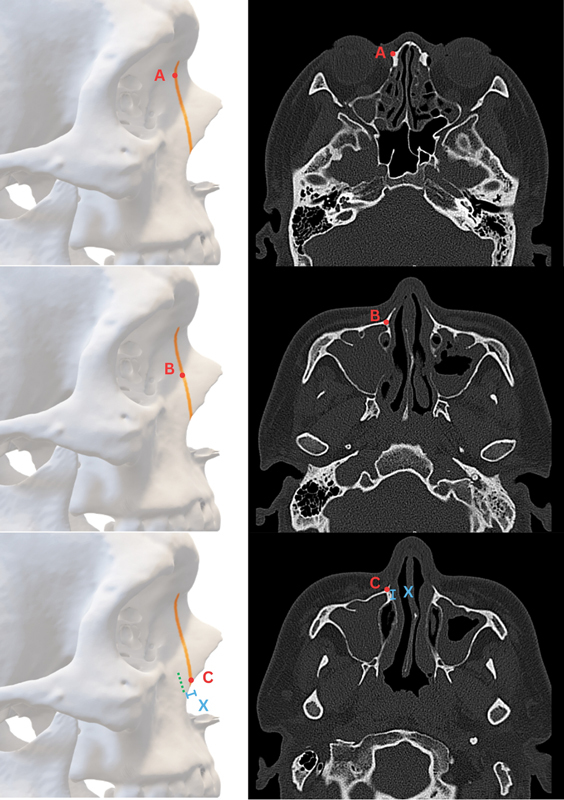
The low-to-low osteotomy path was defined using three CT points: It began at the lacrimal fossa (corresponding to the area of the medial canthal ligament) (A) and passed through the widest point of the nose (B). The osteotomy line was projected to a third point over the most caudal nasal bone (C), and the distance from this third point to the inferior turbinate was then measured (X). (A) Lacrimal fossa, (B) the widest point of the nose at the frontal process of the maxilla, (C) the projected osteotomy line over the most caudal nasal bone, and (X) distance from the projected osteotomy line to the inferior turbinate.

The classic lateral osteotomy extends from the nasofrontal groove toward the medial canthus, passing slightly anterior to the nose's widest point. 8 The low-to-low osteotomy path was defined using three CT points: It began at the lacrimal fossa (corresponding to the area of the medial canthal ligament) and passed through the widest point of the nose (at the frontal process of the maxilla on axial slices). The osteotomy line was projected to a third point over the most caudal nasal bone, and the distance from this third point to the inferior turbinate was then measured ( Fig. 1 ).

### Radiological Measurements: Nasal Characteristics

**Fig. 2:**
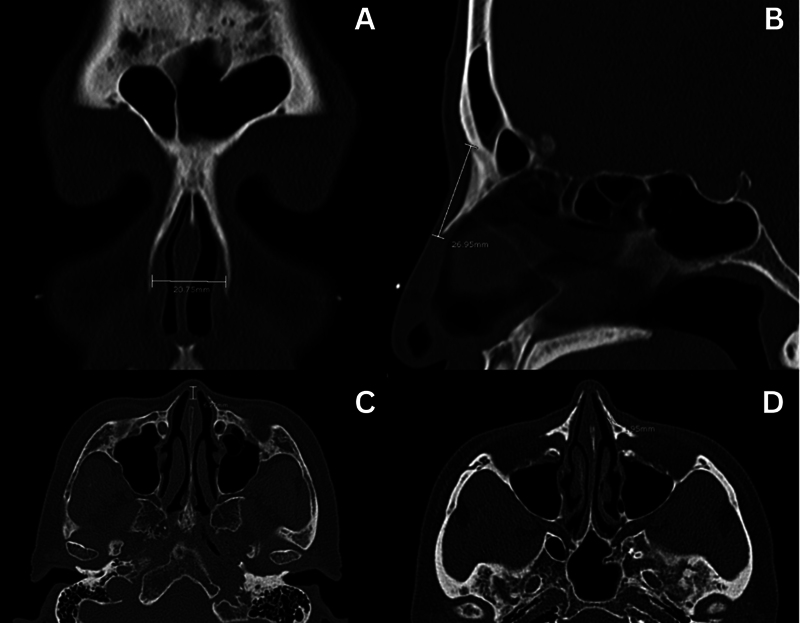
( **A** ) Width of piriform aperture, ( **B** ) length of nasal bone, ( **C** ) rhinion–nasomaxillary suture distance, and ( **D** ) nasal bone thickness.

The nasal characteristics assessed included lateral nasal bone thickness, nasal bone length, pyriform aperture width, and rhinion–nasomaxillary suture distance ( Fig. 2 ). Lateral nasal bone thickness was measured on both sides of the lateral osteotomy path, just anterior to the widest point of the nose but posterior to the nasomaxillary suture. The nasal bone length was measured from the frontonasal suture to the endpoint of the nasal bone in a sagittal reformatted image. The width of the pyriform aperture was measured at its widest points between the inferior borders of the nasal bone in the coronal reformatted image. Rhinion–nasomaxillary suture distance was measured as the length from the rhinion to the nasomaxillary suture in the axial reformatted image. 9

### Statistical Analysis

All data were analyzed using STATA software version 14.0 (StataCorp LLC, College Station, TX). Continuous data were reported as means with standard deviations, while categorical data were reported as frequencies. Pearson's correlation and linear regression analyses were used to evaluate relationships between the distance from the inferior nasal turbinate to the lateral low-to-low osteotomy path and nasal characteristics, with a significance threshold of *p* < 0.05. Intraclass correlation coefficient (ICC) from a two-way mixed-effects model was used to evaluate intrarater reliability.

## Results

**Fig. 3:**
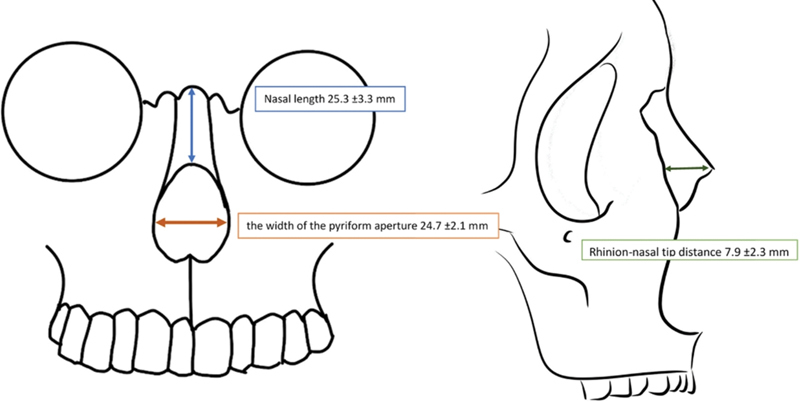
Demonstrates nasal characteristics, including nasal bone length, width of pyriform aperture, and rhinion–nasomaxillary suture distance.

Eighty-one patients participated in the study ( Table 1 ). Their ages ranged from 20 to 83 years, with a mean age of 46.7 ± 14.9 years. Of these patients, 35 were female (43.2%), and 46 were male (56.8%). The mean distance between the inferior turbinate and the lateral low-to-low osteotomy path on the right side was 7.0 ± 3.2 mm (min 0.6 mm, max 16.6 mm), and 6.5 ± 3.0 mm (min 0.4 mm, max 15.4 mm) on the left side ( Table 1 ). None of the 81 patients in this study showed an intersection between the anterior nasal turbinate and the lateral low-to-low osteotomy line. The mean nasal sidewall thickness was 1.8 ± 0.5 mm on both sides. The mean nasal bone length was 25.3 ± 3.3 mm, and the pyriform aperture width averaged 24.7 ± 2.1 mm. The mean rhinion–nasomaxillary suture distance was 7.9 ± 2.3 mm ( Table 1 and Fig. 3 ).

**Table 1:** Demographic and anatomical characteristics of the study population ( *n* = 81)

Category	Parameter	Value
Demographics	Gender (male), *n* (%)	46 (56.8%)
	Gender (female), *n* (%)	35 (43.2%)
	Age (years), mean ± SD	46.7 ± 14.9
Osteotomy path	Distance to inferior turbinate (left), min, max, mean ± SD	0.4, 15.4, 6.5 ± 3.0 mm
(Virtual simulation)	Distance to inferior turbinate (right), min, max, mean ± SD	0.6, 16.6, 7.0 ± 3.2 mm
Average distance, min, max, mean ± SD mean ± SD	0.5, 16.0, 6.7 ± 3.0 mm
Nasal anatomy	Nasal bone thickness (left), mean ± SD	1.8 ± 0.5 mm
	Nasal bone thickness (right), mean ± SD	1.8 ± 0.5 mm
	Length of nasal bone, mean ± SD	25.3 ± 3.3 mm
	Width of pyriform aperture, mean ± SD	24.7 ± 2.1 mm
	Rhinion–nasomaxillary suture distance, mean ± SD	7.9 ± 2.3 mm

Univariate linear regression was conducted to examine the relationship between nasal characteristics (nasal bone length, pyriform aperture width, and rhinion–nasomaxillary suture distance) and the distance between the inferior nasal turbinate and the lateral low-to-low osteotomy path ( Table 2 ). No statistically significant correlation was found between the distance from the inferior nasal turbinate to the osteotomy path and the nasal bone length or pyriform aperture width. However, there was a significant inverse correlation between the rhinion–nasomaxillary suture distance and the result ( *r* = −0.269, *p* = 0.014).

**Table 2:** Variables that affect the distance from the inferior nasal turbinate to lateral low-to-low nasal osteotomy path

Variables	Osteotomy distance	Nasal bone length	Width of the pyriform aperture	Rhinion–nasomaxillary suture distance
Osteotomy distance	1	−0.031 ( *p* = 0.782)	0.028 ( *p* = 0.797)	− **0.269 (0.014)**
Nasal bone length	–	1	−0.106 ( *p* = 0.342)	**0.275 (** ***p*** ** = 0.013)**
Width of the pyriform aperture	–	–	1	−0.184 ( *p* = 0.098)
Rhinion–nasomaxillary suture distance	–	–	–	1

**Fig. 4:**
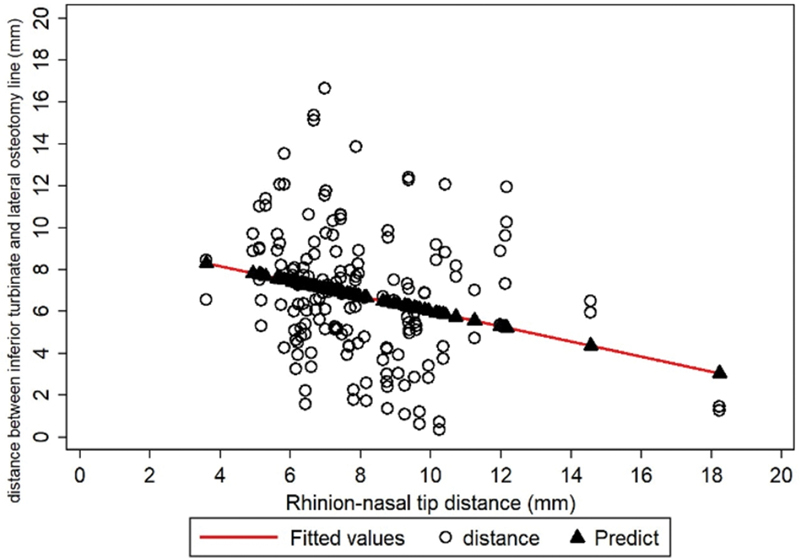
Scatter plot showing the correlation between the inferior nasal turbinate and the lateral low-to-low nasal osteotomy path and rhinion–nasomaxillary suture distance. (X) Rhinion–nasomaxillary suture distance (mm), (Y) distance between the inferior nasal turbinate and the lateral low-to-low nasal osteotomy path (mm).

A linear regression model was used to examine the relationship between the rhinion–nasomaxillary suture distance and the distance between the inferior nasal turbinate and the lateral low-to-low osteotomy path ( Table 3 and Fig. 4 ). The distance was found to have a significant inverse relationship with the outcome (β = −0.35, 95% CI: −0.56 to −0.15, *p* = 0.001, the coefficient of determination [ *R* ^2^ ] = 0.067). This suggests that for each 1 mm increase in rhinion–nasomaxillary suture distance, the result is predicted to decrease by 0.35 mm, holding all other variables constant. The intercept (constant) was 9.60. The regression line equation was as follows:

**Table 3:** Distance between the inferior nasal turbinate and the lateral low-to-low nasal osteotomy path

Variables	Coef. (95% CI)	*p* -Value
Distance		
Rhinion–nasomaxillary suture distance	−0.35 (−0.56 to −0.15)	0.001
Constance(a)	9.60	

Abbreviations: CI, confidence interval; Coef., coefficient.

y, Osteotomy distance; x, rhinion–nasomaxillary suture distance; a, constance.

Linear regression model y = a + bx.

**Table:** 

y, Distance between inferior turbinate and lateral osteotomy line (mm)	x, Rhinion–nasomaxillary suture distance (mm)								
	1	3.3	5.6	7.9	10.2	12.5	14.8	17.1	19.4
y = a + bx = 9.60 + (−0.35) rhinion–nasomaxillary suture distance	9.25	8.45	7.64	6.84	6.03	5.23	4.42	3.62	2.81

Distance between the inferior nasal turbinate and the lateral low-to-low osteotomy path = 9.60 + (−0.35 × rhinion–nasomaxillary suture distance)

To evaluate intrarater reliability, all measurements were repeated by the same observer after 3 months. The intrarater reliability of the measurements was excellent, with a high degree of consistency. The ICC from a two-way mixed-effects model showed excellent reliability for single measurements (ICC = 0.98) and average measurements (ICC = 0.99; Supplementary Table S1 [available in the online version only]).

## Discussion

With the increasing prevalence of rhinoplasty, studies on nasal anatomy have become more refined. Modern technologies like CT scans provide more precise anatomical visualization than previously available methods. Webster's triangle, first described by Webster et al, is a triangular bony area at the pyriform aperture that should be avoided during low lateral osteotomy to prevent airway narrowing and complications. This triangular area is defined externally by the superior border of the pyriform aperture and internally by the head of the inferior nasal turbinate. 4

Traditionally, to avoid narrowing the nasal base and airway, curved or angulated lateral osteotomy lines have been recommended. 4 However, recent studies suggest that avoiding Webster's triangle may not be necessary, as no medial displacement of the inferior turbinate head has been observed with lateral low-to-low osteotomy. 1 2 4 Despite being invasive and irreversible, nasal osteotomy must be performed with high precision, and executing a straight-line osteotomy can be easier to control than a curved or angulated line.

In this study, a lateral low-to-low osteotomy path was recreated on CT scans according to the methodology described by Tebbetts. 8 The lateral low-to-low osteotomy began at the medial canthus and extended linearly to the widest part of the nasal bone, continuing to the pyriform aperture. As shown in 3D images, none of the 81 patients had their anterior nasal turbinate intersect with the lateral low-to-low osteotomy line. This finding suggests that lateral low-to-low osteotomy can be safely performed without regard to Webster's triangle. However, it is important to note that decreased airflow is still expected due to the narrowing of the airway surface around the internal valve. 6 To maintain broader implications, further clinical research using airway resistance measurements is recommended to bridge the gap between anatomical distance and functional outcomes.

Further analysis using linear regression revealed a significant negative relationship between the distance from the inferior nasal turbinate to the lateral low-to-low osteotomy path and the rhinion–nasomaxillary suture distance, which reflects nasal dorsum projection. For every 1 mm increase in rhinion–nasomaxillary suture distance, there was a corresponding decrease in the distance between the inferior nasal turbinate and the osteotomy path. In clinical practice, a low-to-low lateral osteotomy can be executed by advancing from the nasofrontal groove toward the medial canthus, passing slightly anterior to the nose's widest point, irrespective of the inferior turbinate's location. Surgeons should exercise greater caution regarding Webster's triangle in patients with high dorsum projection compared with those with flat nasal bones, aligning with findings by Guyuron 6 that shorter nasal bones experience less airway narrowing after lateral osteotomy. However, airway narrowing may still result from nasal bone medialization even in the absence of inferior turbinate involvement. Further clinical studies are needed with more validated outcomes, using airway resistance measurements and endoscopic findings.

Few studies have investigated racial differences in nasal bone characteristics. 10 Lee et al evaluated the nasal bone length and pyriform aperture width in 75 South Korean subjects using 3D CT scans, finding that the lateral osteotomy thickness was 2.03 ± 0.35 mm, nasal bone length was 20.95 ± 5.99 mm, and pyriform aperture width was 24.01 ± 2.34 mm. 9 Karadag et al examined 80 Anatolian adults and found a nasal bone thickness of 1.85 ± 0.32 mm in males and 1.91 ± 0.46 mm in females, a nasal bone length of 30.61 ± 1.26 mm in males and 29.01 ± 1.12 mm in females, and a pyriform aperture width of 18.83 ± 2.17 mm in males and 18.19 ± 1.85 mm in females. 11 Our findings, comparing Thai nasal characteristics, align more closely with those from the South Korean study. 10 12

The strengths of this study include its novelty, as it is the first to report nasal characteristics specific to Thai patients, and its insights into the correlation between the distance from the inferior nasal turbinate to the lateral low-to-low osteotomy path and the rhinion–nasomaxillary suture distance.

This study has several limitations. First, the findings are based on a CT-based virtual simulation of the lateral low-to-low osteotomy line. As a static anatomical model, it represents a simulated path and does not account for intraoperative fracture patterns, soft-tissue displacement, or postoperative airway function. Consequently, while the study indicates anatomical clearance, it does not demonstrate functional airway safety. Additionally, the study population consisted solely of Thai patients. While Thai anatomy is often grouped within the broader East Asian category, the distinct anatomical variations between Southeast and Northeast Asian populations may limit the generalizability of these findings to all East Asian subgroups.

### Conclusion

This study provides new insights into the nasal characteristics of Thai patients and offers evidence supporting the safety of lateral low-to-low osteotomy without regard to Webster's triangle. The significant relationship between the rhinion–nasomaxillary suture distance and the distance from the inferior nasal turbinate to the lateral low-to-low osteotomy path suggests that surgeons should be cautious when performing osteotomies in patients with high nasal dorsum projection. Further research is needed to explore these findings in other racial populations.
