# Supplementary material for: Anatomical Relationship of Lateral Low-to-Low Nasal Osteotomy and Inferior Nasal Turbinate. Is Webster's Triangle Still Important?
Source: Arch Plast Surg. 2026 May 29;53(3):227–33. doi: 10.1055/a-2836-2147 (PMC13287923; doi:10.1055/a-2836-2147)
Supplement: Supplementary file 1 — Supplementary Material [file 10-1055-a-2836-2147-s25jul0105oa.pdf]

variable	ICC(95%CI)
Left distance between inferior turbinate and lateral osteotomy line	
Individual	0.98(0.96 to 0.99)
Average	0.99(0.98 to 0.99)
F(19, 38) = 148.38, p= 0.000	
Right distance between inferior turbinate and lateral osteotomy line	
Individual	0.96(0.92 to 0.98)
Average	0.98(0.97 to 0.99)
F(19, 38) = 77.25, p= 0.000	
Nasal bone length	
Individual	0.99(0.97 to 0.99)
Average	0.99(0.99 to 1.00)
F(19, 38) = 242.74, p= 0.000	
Width of pyriform aperture	
Individual	0.90(0.81 to 0.96)
Average	0.96(0.92 to 0.98)
F(19, 38) = 29.99, p= 0.000	
Rhinion-nasal tip distance	
Individual	0.95(0.90 to 0.98)
Average	0.98(0.96 to 0.99)
F(19, 38) = 61.14, p= 0.000	
